# Supplementary material for: Environmental DNA reveals patterns of biological invasion in an inland sea
Source: PLoS One. 2023 Dec 27;18(12):e0281525. doi: 10.1371/journal.pone.0281525 (PMC10752502; doi:10.1371/journal.pone.0281525)
Supplement: S1 File — (DOCX) [file pone.0281525.s001.docx]

**Supplement 1.**

[**https://github.com/ramongallego/eDNA.and.Ocean.Acidification.Gallego.et.al.2020**](https://github.com/ramongallego/eDNA.and.Ocean.Acidification.Gallego.et.al.2020)

A Github repository containing links to raw FASTA sequence data, as well as sequencing quality control, and taxon assignment to ASVs.

[**https://github.com/jdduprey/patterns_of_invasion**](https://github.com/jdduprey/patterns_of_invasion)

A Github repository containing non-native species data processing, quality control, and analysis.

[**https://github.com/jdduprey/patterns_of_invasion/blob/main/data/introduced_species_BLASTn.csv**](https://github.com/jdduprey/patterns_of_invasion/blob/main/data/introduced_species_BLASTn.csv)

A CSV file containing BLASTn output for all candidate non-native species ASVs. This includes the top 5 sequence matches, query accession, sequence ID, percent identity, scientific name and other relevant data.


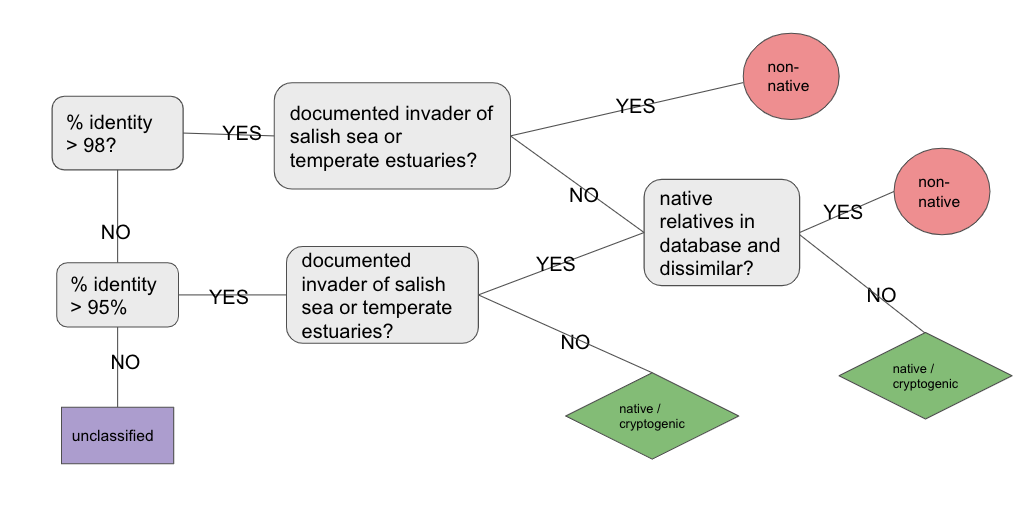


**Fig S1.** Flowchart of the decision process for classification of an ASV as either *non-native*, *native/cryptogenic,* or *unclassified*. *Unclassified* here indicates that we were not able to confidently match the ASV to a species level assignment. If no peer-reviewed source contained the species distribution, or if there were fewer than three published detections of the species, then the ASV was classified as belonging to a *cryptogenic* species.
