# Supplementary material for: Environmental DNA reveals patterns of biological invasion in an inland sea
Source: PLoS One. 2023 Dec 27;18(12):e0281525. doi: 10.1371/journal.pone.0281525 (PMC10752502; doi:10.1371/journal.pone.0281525)
Supplement: Attachment — Submitted filename: comments.docx [file pone.0281525.s002.docx]

The study entitled “Environmental DNA reveals patterns of biological invasion in an inland sea” study which habitat types are most prone to biological invasion and what are the vector that drive those invasions. I am not an expert on environmental DNA but I think that the paper is written in a clear manner and it is easy to follow the flow of the research article. Beside that, I’ve made some suggestion that, in my opinion, can strength the paper.

I think that in some part this paper can be improve with some work on the images. In the detailed comments I have made different advice.

To strengthen the paper, I suggest:

1. To Improve the method part as suggested in the detailed comments.
2. To improve the readability of the figure created.

DETAILED COMMENTS:

- L123-125 repetition of concept, I suggest rewrite the sentence to make it clearer.
- L186-188 Is there any reference on this approach used? Any previous work?
- L202 you should cite both R and the R package used.
- L211 cite the vegan package.
- Figure 1 is missing the scale and the northern arrow. I also suggest using different symbol instead of colors to point the study sites.
- Figure 5 is it possible to have symbols instead of colors? The graph with color is pretty hard to read.
- Figure 6 if you use color to define the study site you can’t change the color you assigned to another figure. For example, at TW is assigned red but in the fig 5 is the blue one. That make the readability of the graph lower.
