# Supplementary material for: Environmental DNA reveals patterns of biological invasion in an inland sea
Source: PLoS One. 2023 Dec 27;18(12):e0281525. doi: 10.1371/journal.pone.0281525 (PMC10752502; doi:10.1371/journal.pone.0281525)
Supplement: Attachment — Submitted filename: response_to_reviewers.docx [file pone.0281525.s003.docx]

We appreciate the comments made by the reviewer and have addressed them as described below.

**L123-125 repetition of concept, I suggest rewrite the sentence to make it clearer.**

I’ve eliminated the repeated concept from the previous paragraph L114-115. I’ve also clarified the comparison of abiotic habitat factors between sites L124-L126.

**L186-188 Is there any reference on this approach used? Any previous work?**

To our knowledge no previously published work has taken this exact approach to classifying species as native, non-native or cryptogenic. Previously published invasive species research often reports similar approaches, or does not explicitly report their classification process. We offer examples from two of the non-native species metabarcoding studies cited in our manuscript:

Lacoursiere-Roussel et al (2018) state that they identified potential “NIS invaders” based on previously published risk assessments, and monitoring studies of their study region – an approach similar to our own. They also highlight that “focal taxa [potential NIS invaders] vary among surveys”, an observation that highlights the challenges of this classification process in general.

Holmen et al (2019) do not explicitly state how they classify species as non-native to their study region. “In total 18 NIS to the study region and 24 species documented as NIS in other regions were detected across the four sites (see Supplementary Table [2](https://www.nature.com/articles/s41598-019-47899-7#MOESM3) for full list). Out of the detected NIS, eight were present in the list of 21 NIS previously detected in rapid assessment (RA) surveys at the sampling sites”.

We note that in the context of the broader non-native species literature our approach to classifying species is conservative – relying on multiple databases sourced from peer reviewed literature, and requiring multiple peer-reviewed sources to have already classified a species as non-native.

**L202 you should cite both R and the R package used.**

Citations added for both.

**L211 cite the vegan package.**

Citation added.

**Figure 1 is missing the scale and the northern arrow. I also suggest using different symbol instead of colors to point the study sites.**

Fig1: I have added a scale bar and a north arrow. I have made the study sites different symbols in addition to different colors to represent that different substrate types.

**Figure 5 is it possible to have symbols instead of colors? The graph with color is pretty hard to read.**

Fig5: I think symbols for each site distract from the main take-away of this scatter plot which is the relationship between temperature and relative non-native richness. I have increased the size of each data-point to make the color more legible.

**Figure 6 if you use color to define the study site you can’t change the color you assigned to another figure. For example, at TW is assigned red but in the fig 5 is the blue one. That make the readability of the graph lower.**

Fig 6: I have changed the colors on Figure 6 so that each site has the same color on both Figure 5 and Figure 6.
